# Combining Physiological and Metabolomic Analysis to Unravel the Regulations of Coronatine Alleviating Water Stress in Tobacco (*Nicotiana tabacum* L.)

**DOI:** 10.3390/biom10010099

**Published:** 2020-01-07

**Authors:** Jiayang Xu, Yuyi Zhou, Zicheng Xu, Zheng Chen, Liusheng Duan

**Affiliations:** 1 College of Agronomy and Biotechnology, China Agricultural University, Beijing 100193, China; meet_jiayangxu@126.com (J.X.); zhouyuyi711@126.com (Y.Z.); 2 College of Tobacco Science, Henan Agricultural University, Zhengzhou 450002, China; zichengxu@henau.edu.cn (Z.X.); chenzhengphd@163.com (Z.C.)

**Keywords:** water stress, reactive oxygen species (ROS), coronatine, phytohormone, metabolic network

## Abstract

Drought is a major abiotic stress that restricts plants growth, development, and yield. Coronatine (COR), a mimic of JA-Ile, functions in plant tolerance to multiple stresses. In our study, we examined the effects of COR in tobacco under polyethylene glycol (PEG) stress. COR treatment improved plant growth under stress as measured by fresh weight (FW) and dry weight (DW). The enzyme activity assay indicated that, under osmotic stress conditions, the activities of superoxide dismutase (SOD), catalase (CAT), ascorbate peroxidase (APX), and glutathione reductase (GR) were enhanced by COR treatment. Histochemical analyses via nitrotetrazolium blue chloride (NBT) and 3,3′-diaminobenzidine (DAB) staining showed that COR reduced reactive oxygen species (ROS) accumulation during osmotic stress. Metabolite profiles revealed that COR triggered significant metabolic changes in tobacco leaves under osmotic stress, and many essential metabolites, such as sugar and sugar derivatives, organic acids, and nitrogen-containing compounds, which might play active roles in osmotic-stressed tobacco plants, were markedly accumulated in the COR-treated tobacco. The work presented here provides a comprehensive understanding of the COR-mediated physiological, biochemical, and metabolic adjustments that minimize the adverse impact of osmotic stress on tobacco.

## 1. Introduction

Drought is an adverse abiotic factor that severely limits plants growth, development, and productivity. With global climate changes and increasing demands for food production, drought stress-related yield losses have received increasing attention in recent years [1,2]. From the stimulated grain crops modeling, more frequency to the arid extreme events are predicted to augment by the end of the twenty-first century, leading to severe water crisis and double the yield reduction in cultivation areas [3,4]. In addition to the decline of production, drought also triggers an array of morphological, physiological, and biochemical responses, including the inhibition of cell elongation and expansion, the disruption of major components of photosynthesis, the triggering of severe oxidative bursts, and even resulting in the death of the plants [5]. Therefore, it is crucial to expound the defense responses through which plants improve tolerance (or resistance) against drought, specifically via the cellular and the molecular mechanism (e.g., regulation of multiple functional genes and proteins, accumulation of diverse stress-associated osmolytes) that meet the needs for growth and development.

Coronatine (COR), a non-host-specific phytotoxin, is produced by *Pseudomonas syringae* pathovars [6]. The biological activity of COR is similar to that of JA-Ile, and both COR and JA-Ile share the same receptor coronatine insensitive 1 (COI1) [7,8]. COR widely takes part in a plant’s biological and biochemical process, including ethylene emission, anthocyanidin production, auxin synthesis, and alkaloid accumulation [8,9,10,11]. Micro-doses of COR have been demonstrated to participate in plants stress tolerance, including tolerance of salinity stress in cotton [12], drought stress in soybean and cauliflower [13,14], heat stress in wheat [15], and chilling stress in cucumber [16]. These studies revealed that COR could enhance plant stress resistance by maintaining enhanced photosynthetic performance and improving antioxidant enzyme activities. More recently, our work deciphered the positive role of COR in relieving osmotic stress in rice plants by using whole-genome transcript analysis [17]. However, there was limited research focusing on the dynamic change of metabolites of coronatine in the case of alleviating water stress in plants. 

Plant metabolites, which comprise a large number of intermediate compounds and products of various metabolic pathways, have vital roles in plants responses to stresses through cellular structure, cell signaling, energy metabolism, and whole-plant resource utilization [18]. Metabolomics is a powerful tool that provides an overview of biological processes by detecting low molecular weight metabolites and is widely used to illuminate the molecular mechanism of plant responses to and defenses against various stressors [19,20]. Using untargeted metabolomic analysis, Scalabrin et al. identified over two hundred metabolites under disparate stresses, such as high temperature, drought, and chromium stress, in both wild and transgenic *Nicotiana langsdorffii* [21]. A recent study with gas chromatography-mass spectrometry in *Solanum aethiopicum* under drought stress identified several carbohydrates and organic acids with the potential to contribute to drought stress tolerance [22]. 

Tobacco (*Nicotiana tabacum* L.) is a commercial-important broadleaf crop with a large planting area in China and accounts for one-third of tobacco production worldwide. However, tobacco is sensitive to drought stress, and water deficits especially occur at the rosette and ripening stages, seriously restricting its growth and yields. In addition to the model plant *Arabidopsis*, tobacco is also used as an excellent model for genetic studies in dicotyledons. Our present work here first detected physiological traits of tobacco pre-treated with COR and performed metabolic profiling analyses to evaluate physiological changes induced by polyethylene glycol (PEG)-simulated drought stress. This integrative analysis highlights the molecular mechanisms of COR-induced osmotic tolerance in tobacco at metabolic levels. 

## 2. Materials and Methods 

### 2.1. Plant Material and PEG Treatment

The tobacco line K326 (*Nicotiana tabacum* L.), which is a representative cultivated tobacco in China, was used for this study. Tobacco seedlings were cultivated in a plant growth chamber with 14 h light (26 °C) and 10 h dark (22 °C). At the four-leaf stage, normally grown tobacco lines were randomly selected for further analysis. Coronatine (COR) was obtained from China Agricultural University. For COR treatment, the prepared COR was diluted with Hoagland nutrient solution to 1 nM. After 24 h of treatment with COR, the corresponding tobacco lines were subjected to 20% polyethylene glycol-6000 (PEG-6000)-simulated osmotic stress (ψs = −0.49 MPa). Thus, our experiment included four treatments: CK, COR, PEG, and PEG+COR. One day after the PEG treatment, all leaf samples from these four treatments were immediately frozen in liquid N_2_ and stored at −80 °C for metabolite analysis. 

### 2.2. Measurement of Fresh Weight (FW) and Dry Weight (DW)

The FW of tobacco seedlings was determined immediately after harvest, and the DW of the whole tobacco plants was measured after drying at 105 °C (1 h) and at 80 °C until constant weight. Five tobacco samples in each replication were examined. 

### 2.3. Determination of Superoxide Anion (O_2_^•−^) and Hydrogen Peroxide (H_2_O_2_) Concentrations

In tobacco leaves at different sampling points, the O_2_^•−^ production speed was measured according to the method of Tian et al. [23]. Briefly, 0.3 g leaf tissue was ground in 2 mL phosphate buffer containing 1% PVP and 0.15 mM EDTA. The homogenate was centrifuged at 13,000× *g* for 20 min at 4 °C, then 0.5 mL supernatant was collected and 0.5 mL phosphate buffer was added, along with 1 mL hydroxylamine chloride, to incubate for 1.5 h at 25 °C. After incubating with 1 mL sulfanilic acid and 1 mL a-naphthylamine, the absorbance was detected at 530 nm. The in situ detection of O_2_^•−^ was performed with nitrotetrazolium blue chloride (NBT) staining following the method of Scarpeci et al. [24]. For the measurement of H_2_O_2_, 0.5 g leaf tissue was ground in liquid nitrogen and then suspended in 5 mL of a solution containing 10 mM phosphate buffer (pH 7.4). The homogenate was centrifuged at 10,000 rpm for 15 min at 4 °C. H_2_O_2_ reacts with molybdenic acid to form a complex compound, which can be measured at 405 nm. The H_2_O_2_ content was determined using a kit (No. A064, Nanjing Jiancheng Bioengineering Institute, Nanjing, China) according to the manufacturer’s instructions. The in situ accumulation of H_2_O_2_ was determined by 3,3′-diaminobenzidine (DAB) staining using the methods described by Daudi et al. [25]. Protein contents were colorimetrically quantified using Coomassie brilliant blue G-250 as described by Bradford with bovine serum albumin (BSA) as a standard [26].

### 2.4. Determination of Antioxidant Enzyme Activities

In brief, 0.5 g leaf tissue was ground in liquid nitrogen and then suspended in 5 mL of a solution containing 10 mM phosphate buffer (pH 7.4). The homogenate was centrifuged at 4000 rpm for 15 min at 4 °C. The activities of superoxide dismutase (SOD, EC 1.15.1.1) and catalase (CAT, EC 1.11.1.6) were measured using assay kits (No. A001 for SOD, No. A007 for CAT, Nanjing Jiancheng Bioengineering Institute, Nanjing, China) according to the manufacturer’s instructions. The activity of SOD was determined by measuring the absorbance at 550 nm, and one unit activity was defined as the amount of SOD are needed to produce 50% inhibition of reduction of 1 mL reaction solution per milligram of tissue protein. The activity of CAT was determined by measuring the absorbance at 405 nm, and one unit activity was defined as the amount of enzyme that causes the decomposition of 1 mmol H_2_O_2_ per second.

For the assay of ascorbate peroxidase (APX, EC 1.11.1.11), 0.5 g leaf tissue was ground in liquid nitrogen and then suspended in 5 mL of a solution containing 10 mM phosphate buffer (pH 7.4). The homogenate was centrifuged at 1000 rpm for 15 min at 4 °C. The activity of APX was measured using assay kits (No. A123, Nanjing Jiancheng Bioengineering Institute, Nanjing, China) according to the manufacturer’s instructions. The activity of APX was determined by measuring the absorbance at 290 nm, and one unit activity was defined as the amount of enzyme that catalyzes a reaction of 1 mmol ASA per milligram of tissue protein.

The activities of glutathione reductase (GR, EC 1.6.4.2) were determined following the method of Li et al. [27]. Briefly, 0.2 g leaf tissue was ground in liquid nitrogen and extracted with 5 mL of a solution containing 50 mM potassium buffer (pH 7.0), 1mM EDTA and 1% PVP. The activity of GR was determined by measuring the change of absorbance at 340 nm due to the oxidation of NADPH.

### 2.5. Sample Preparation for LC-MS

The freeze-dried tobacco leaves were ground to a uniform powder, and 100 mg powder of each sample was macerated with 1 mL of methanol-acetonitrile (1:1, *v*/*v*). Then, the homogenate was sonicated for 30 min (4 °C). After centrifuging at 14,000× *g* for 20 min (4 °C), the supernatant was collected for LC-MS analysis.

### 2.6. UHPLC-Q-TOF/MS Conditions

A UHPLC system (1290 Infinity LC, Agilent Technologies, Santa Clara, CA, USA), with the addition of a quadrupole time-of-flight mass spectrometer (Triple TOF 6600, AB Sciex, Foster City, CA, USA) with an electrospray ionization source (ESI), was used for the analysis. The samples were separated on an ACQUITY UPLC BEH 1.7 μm column (2.1 mm × 100 mm, Waters, Ireland). The mobile phase, which contained A (containing 25 nM ammonium acetate and 25 nM ammonium hydroxide in water) and B (acetonitrile), was used for both of the positive and negative ion modes. The gradient was set as follows: 95% B (1 min), which decreased linearly to 65% (13 min) and then decreased linearly to 40% (2 min); and 40% B (3 min), which then increased to 95% (0.1 min). The ESI parameters were as follows: ion source gas 1, 60; ion source gas 2, 60; and ion spray voltage floating, ± 5500 V. With respect to the MS-only acquisition, apparatus were set to a *m*/*z* range of 60–1000 Da and the scanning of TOF MS was 0.2 s per spectrum. For the auto MS/MS acquisition, apparatus were set to a *m*/*z* range of 25–1000 Da and the with scanning of product ion was 0.05 s per spectra. For the product ion scan acquisition: fixed collision energy, 35 V ± 15 eV; declustering potential, 60 V (+) and −60 V (−); and exclusion of isotopes within 4 Da.

### 2.7. Data Analysis

For the assay of FW, DW, O_2_^•−^ production speed, H_2_O_2_ content, activities of antioxidant enzymes, significant differences between CK, COR, PEG, and PEG+COR treatment were assessed by one-way ANOVA with LSD post hoc tests, performed by SPSS (19.0, IBM Corp, Armonk, NY, USA).

With respect to the metabolomics, raw data files obtained from UHPLC-Q-TOF/MS were transformed to MzXML files by using ProteoWizard MSConvert (version 3.0.913) and XCMS software (version 1.52.0) [28,29,30]. By using XCMS software, the nonlinear alignment, automatic integration, extraction of the peak intensities, peak alignment, and data filtering were finished. All the metabolites were identified by accuracy mass (< 25 ppm) matching and secondary mass spectrogram matching, and the score cutoff was set as 0.8 [31]. MetaboAnalyst 4.0 was used for statistical analysis [32]. Principle component analysis (PCA) and partial least squares discrimination analysis (PLS-DA) were performed for unsupervised multivariate statistical analysis and to identify the most important variables, respectively. The variable importance in projection (VIP) > 1 is perceived as significant in the PLS-DA model [33]. For univariate analysis, the metabolites were detected via a t-test between non-treated and COR-treated tobacco samples, where a *p* value less than 0.05 (*p* < 0.05) was recognized as statistically significant. The significantly changed metabolites were mapped to the KEGG database for understanding the chemical and metabolic pathways.

## 3. Results

### 3.1. Morphological Changes of Seedlings

**Figure 1:**
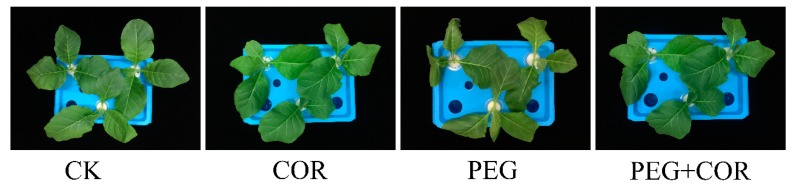
The morphological traits of tobacco treated with coronatine (COR) under normal and osmotic conditions. CK, normal growth conditions; COR, pretreatment with 1 nM COR; polyethylene glycol (PEG), osmotic stress induced by PEG without COR treatment; PEG+COR, pretreatment with 1 nM COR under PEG stress.

From the experimental images of tobacco seedlings under polyethylene glycol (PEG) stress, remarkable differences in morphology were observed in different treatments (Figure 1). PEG-simulated osmotic stress caused leaf wilting in the CK samples, while seedlings treated with COR showed better morphology. Additionally, this could be seen with the recorded data of plant weight. The DW of seedlings was significantly reduced by 16.86% (Table 1); the FW of COR-treated tobacco markedly increased 25.02% under PEG treatment. However, COR treatment had no significant effects on morphology and plant biomass under well-watered conditions.

### 3.2. Hydrogen Peroxide (H_2_O_2_) and Superoxide (O_2_^•−^) Concentrations

**Figure 2:**
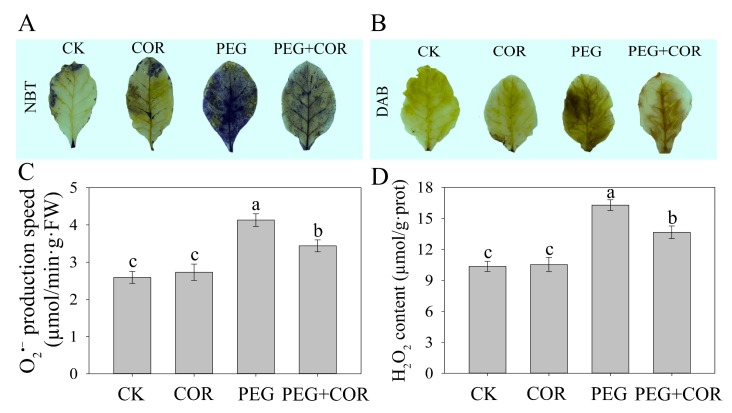
Effect of coronatine on reactive oxygen species (ROS) content in leaves of tobacco seedlings under osmotic conditions. In situ detection of O_2_^•−^ by nitrotetrazolium blue chloride (NBT) staining (**A**), in situ detection of H_2_O_2_ by 3,3′-diaminobenzidine (DAB) staining (**B**), the generation of O_2_^•−^ (**C**) and the content of H_2_O_2_ (**D**) in tobacco leaves. Values with bars are the means ± SDs (n = 5), and different letters indicate significant differences at *p* < 0.05.

Osmotic stress-induced oxidative damage usually results in the accumulation of O_2_^•−^ and H_2_O_2_. We, therefore, detected O_2_^•−^ and H_2_O_2_ localization in the CK and COR-treated tobacco plants using NBT and DAB staining, respectively. Under normal conditions, the results of both NBT and DAB staining indicated that a small area contained ROS (Figure 2A,B). However, after 24 h of PEG stress, the leaves of the control tobacco were extensively stained with both NBT and DAB, while few increases in the staining area of ROS were detected in the leaves of the COR-treated plants, showing that under osmotic stress, tobaccos pretreated with COR accumulated less O_2_^•−^ and H_2_O_2_ than the control plants. The quantitative assay of the formation of O_2_^•−^ production and H_2_O_2_ also supported the NBT and DAB staining results, which indicated a significant difference between control and COR-treated samples under PEG-simulated osmotic stress (Figure 2C,D).

### 3.3. Activities of Antioxidant Enzymes

**Figure 3:**
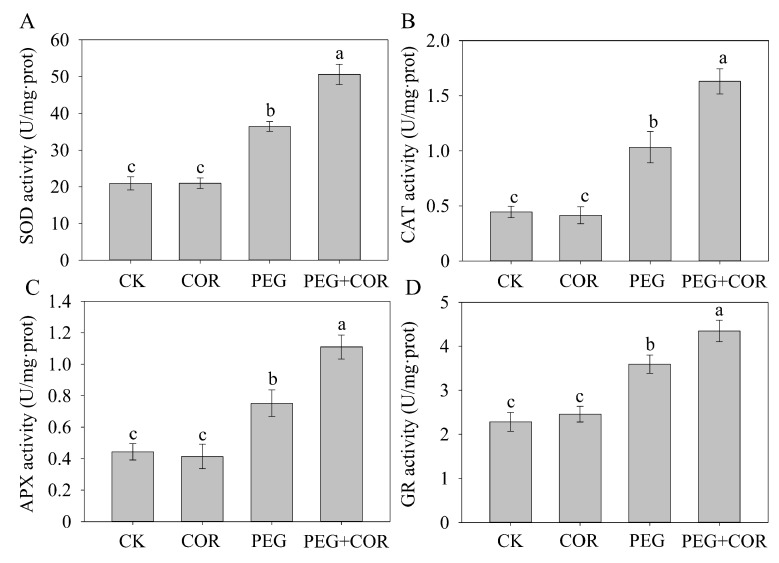
Effect of COR on the activities of superoxide dismutase (SOD) (**A**), catalase (CAT) (**B**), ascorbate peroxidase (APX) (**C**), and glutathione reductase (GR) (**D**) under osmotic conditions. Values with bars are the means ± SDs (n = 5), and different letters indicate significant differences at *p* < 0.05.

To explore the mechanism of COR-mediated osmotic stress tolerance in tobacco, we detected the activities of antioxidant enzymes during different conditions. The exogenous application of COR had little influence on the activities of SOD, CAT, APX, and GR under normal growth conditions (Figure 3). Osmotic-exposed plants exhibited higher activities of these defensive enzymes in all tobacco samples; however, COR-treated tobacco plants exhibited 25.36%, 27.33%, 47.61%, and 20.91% greater SOD, CAT, APX, and GR activities in response to PEG stress, respectively.

### 3.4. Metabolite Profiling

**Figure 4:**
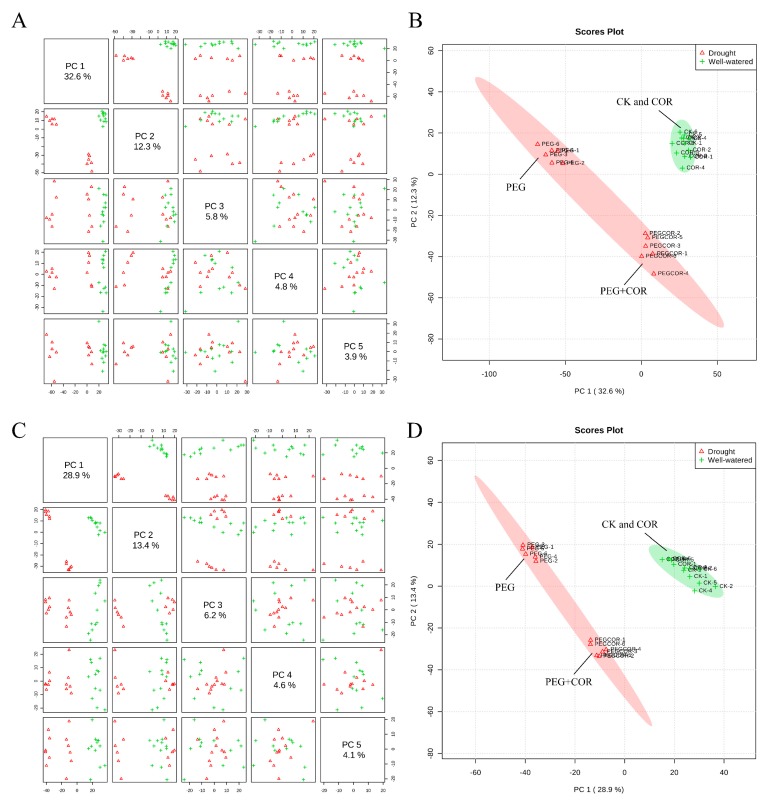
Principal component analysis and 2D score plot of metabolites in tobacco leaves between COR and control treatments in the positive ion mode (**A**,**B**) and negative ion mode (**C**,**D**). CK, normal conditions without COR and PEG treatment; COR, treatment with 1 nM COR; PEG, osmotic stress induced by PEG without COR treatment; PEG+COR, pretreatment with 1 nM COR under PEG stress.

To evaluate regulations of metabolic homeostasis in tobaccos responding to osmotic stress, the metabolite profiles of the tobacco leaves in the control and COR treatments under well-watered and PEG conditions were analyzed using an LC/MS platform by a professional laboratory (Applied Protein Technology, Shanghai, China) [34]. The PCA was performed for normal growth conditions and osmotic stress on both the control and the COR-treated tobacco seedlings (Figure 4). The first component (PC1) and second component (PC2) explained the 32.6% and 12.3% of total variation in the positive ion mode (Figure 4A,B), whereas the PC1 and PC2 in the negative ion mode explained the 28.9% and 13.4% of the variation (Figure 4C,D). The score plot displayed two different groups, which were associated with well-watered and PEG-stressed samples at corresponding sampling time point, indicating a distinct separation of the metabolite profiles under the two conditions. The PC1 mainly explained the separation of samples caused by PEG stress, whereas PC2 mainly explained divergence between COR-treated and non-treated tobacco plants. The control and COR-treated tobacco seedlings were separated from each other under osmotic stress, whereas these treatments overlapped under well-watered conditions.

To identify the significantly changed metabolites between COR-treated and non-treated tobacco seedlings under normal growth conditions and PEG stress, a PLS-DA and a univariate analysis were carried out (Figure S1). The PLS-DA method was able to identify the most important metabolites on account of the variable importance in projection (VIP) scores using a five-component model. We adjusted for multiple testing using VIP > 1, fold change (FC) > 1.5 or < 0.67, and *p* < 0.05 as the criteria to classify the significantly changed metabolites between the COR and control treatments under the two conditions. A total of 6 and 28 metabolites were identified under normal and PEG conditions, respectively (Table 2). Most of the sugars and sugar derivatives (including allose, galactinol, glucose, fructose, mannose, quinovose, maltitol, and myo-inositol) and organic acids (including α-ketoglutarate, chlorogenic acid, citrate, glyceric acid, and quinic acid) were significantly accumulated in tobacco leaves under both well-watered and PEG conditions; however, amino acids, such as arginine, asparagine, histidine, isoleucine, leucine, lysine, norleucine, threonine, and tryptophan, exhibited enhanced accumulation under osmotic stress in control plants compared with the accumulation in COR plants. The changes in these metabolites provide important clues for research into the COR-induced specific metabolites in tobacco.

### 3.5. KEGG Metabolic Pathway Analysis of Significantly Changed Metabolites

**Figure 5:**
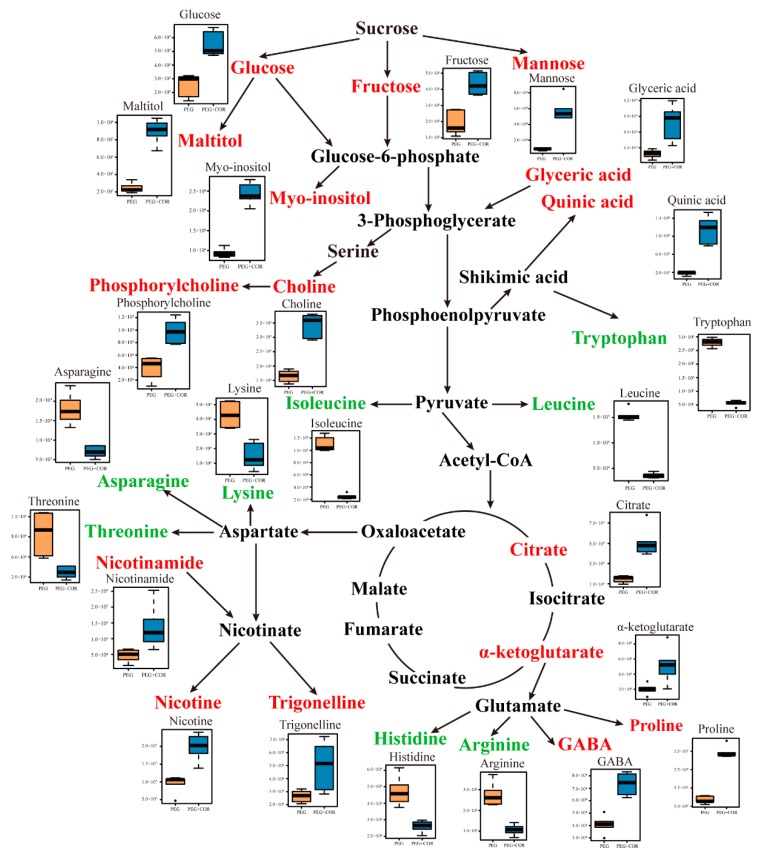
Summary scheme showing the main COR-induced metabolic changes under osmotic stress. The metabolites with red/green color indicate up-regulation/down-regulation in response to COR. The relative abundance of metabolites in different treatment under PEG was shown as box plots.

The metabolites that were significantly different between the tobacco leaves under different conditions (well-watered and PEG) were screened into the KEGG database to detect the biochemical and metabolic pathways that are involved in the response to COR. A total of 12 pathways were enriched based on KEGG plants metabolic pathways, with more than two significantly changed metabolites in one pathway (Table 3). As shown in Table 3, these enriched pathways were mainly involved in the metabolism of carbohydrates (e.g., “galactose metabolism”, “fructose and mannose metabolism”), organic acids (e.g., “citrate cycle”), and amino acids (e.g., “glycine, serine, and threonine metabolism”; “valine, leucine, and isoleucine biosynthesis”; “alanine, aspartate, and glutamate metabolism”). In addition, the significantly changed metabolites and their regulations under PEG stress were shown as Figure 5.

## 4. Discussion

### 4.1. Exogenous COR Improves ROS Scavenging in Plants by Stimulating Antioxidant Enzymes

Drought stress typically triggers the generation of ROS, such as O_2_^•−^ and H_2_O_2_, which can diffuse across the cell membrane and cause cell damage in plants, by the oxidative reaction process of mitochondrial respiration [35,36]. In the present study, in vivo detection of O_2_^•−^ and H_2_O_2_ via both NBT and DAB staining and quantitative assays indicated an obviously increased accumulation of O_2_^•−^ and H_2_O_2_ in tobacco leaves under osmotic stress in the control samples compared the accumulation in the leaves in the COR treatment. The internal protective enzyme-catalyzed clean-up systems in plants help to scavenge ROS and ensure the maintenance of cellular functions. For example, SOD plays a key role in defending against ROS-mediated oxidative injury in plants through dismutation of O_2_^•−^ to O_2_ and H_2_O_2_, whereas CAT directly scavenges H_2_O_2_ by reducing H_2_O_2_ to H_2_O and O_2_. APX is a crucial enzyme involved in the water-water and ascorbate-glutathione (AsA-GSH) cycles; APX catalyzes the reaction of H_2_O_2_ into H_2_O and utilizes AsA as the electron donor. Additionally, GR is a vital constituent of the AsA-GSH cycle and takes part in ROS detoxification via maintaining GSH in its reduced state [37]. Consistent with the results of previous studies of COR-induced stress tolerance in plants [38,39], our results indicated that the positive role of COR-mediated tobacco seedlings to relieve oxidative injury involves improvement in antioxidant enzyme activities.

### 4.2. Exogenous COR Promotes the Accumulation of Carbohydrates Under PEG Conditions

The accumulation of sugars is an essential strategy with which plants withstand abiotic stress since sugars can act as important compatible solutes and signaling molecules in plant defense responses [40]. Moreover, the accumulations of sugars under adverse conditions build blocks for osmoprotectants and provide an energy source for physiological growth by stabilizing the macromolecules [41]. A significantly-enhanced level of carbohydrates, such as glucose, fructose, mannose, quinovose, maltitol, and myo-inositol, was observed in tobaccos pretreated with COR in response to PEG stress. Glucose and fructose are two of the main reducing sugars that are responsible for plant signal transduction to modulate drought tolerance [42]. Elevated level of glucose and fructose is involved in turgor maintenance [43] and associated with improved stress tolerance in many plants [44,45,46]. Mannose has been shown to be an important compatible osmolyte and play a role in protein glycosylation in plants [47]. Mannose also provides efficient precursors for ascorbate synthesis, known as an important ROS-scavenging antioxidant in stress resistance [48,49]. Li et al. revealed that *Agrostis stolonifera* treated with ABA induced significant increase in mannose content under unirrigated conditions [47]. Myo-inositol is the most abundant form of naturally existence isomers of inositols and imparts abiotic stress tolerance to plants. It also serves as the precursor of the synthetic route of the galactinol and raffinose family oligosaccharides (RFO), which may act as sugar signals in abiotic stresses, like glucose and fructose [50]. The increased level of myo-inositol may combat PEG-simulated osmotic stress due to its hydroxyl groups, which can generate a sphere of hydration around macromolecules [51]. Previous evidences have shown that COR could promote soluble sugar content in *Brassica oleracea* L. during water stress [14], and the various carbohydrates with multiple functions identified here in COR-treated plants may interact with plant hormones and many other metabolic processes to regulate osmotic balance and protect the cellular structure from ROS damage under osmotic stress.

### 4.3. Exogenous COR Enhances the Accumulation of Organic Acids Under PEG Stress

Our study noted the accumulation of organic acids, namely, citrate, α-ketoglutarate, glyceric acid, and quinic acid, in tobacco leaves by COR treatment when exposed to osmotic stress. Organic acids not only function as important intermediates in the energy balance and flow in plants but also participate in plant adaption to abiotic stress. The tricarboxylic acid (TCA) cycle (also known as the Krebs cycle) is a vital cellular cycle that drives energy production and provides a carbon skeleton for the biosynthesis of some amino acids. Citrate, the first intermediate involved in the TCA cycle, is known to participate in plants responses to abiotic stress. The increase in citrate accumulation was observed in cotton under drought [52] and tomato under chilling treatment [53]. Wu et al. demonstrated that citrate has the anti-oxidant capacity to modulate redox signaling through scavenging free radicals and chelating iron ions [54]. Moreover, Sung et al. revealed a tissue-specific accumulation of α-ketoglutarate in tomato under nutrition-deficient conditions [55]. α-ketoglutarate serves as the precursor of the glutamate and GABA pools. The upregulation of intermediates in the TCA cycle may reflect acceleration of the TCA pathway, suggesting that COR treatment could facilitate the flow of carbon from glycolysis to manufacture other defense compounds and produce more additional energy, such as NADH and ATP, to address oxidative stress. In addition, glyceric acid, a component of glycolysis, was associated with a greater ability to absorb nutrients and water of wheat under drought conditions [4]. The founding of Li et al. indicated that exogenous β-sitosterol promoted the accumulation of glyceric acid contributing to improved drought tolerance in white clover [56]. These results implied that COR-mediated tolerance to osmotic stress is associated with organic acids belonging to both intermediates and non-intermediates involved in the TCA cycle.

### 4.4. Increases in Some Nitrogen-Containing Metabolites Induced by COR Contribute to Osmotic Resistance

The accumulation of nitrogen-containing compounds induced by COR suggested a potential mechanism by which COR confers osmotic tolerance to tobacco. Nicotinamide is an essential component of pyridine dinucleotide coenzymes NAD(P)H, acting as stress signal and playing defensive roles in many enzymatic oxidation-reduction reactions [57]. It is reported that nicotinamide has a promotive effects on carbohydrate production and protein synthesis when sorghum is exposed to salt stress [58]. Nicotinamide also regulates the content of stress-associated metabolites, such as trigonelline and nicotine. Trigonelline is shown to accommodate cell cycle, stabilize enzyme activity, and modify membrane transport properties [59,60]. Besides these physiological functions, trigonelline also acts as a signal in response to salinity and a water deficit [61,62]. Nicotine is the main form of alkaloid in cultivated tobacco that defends against insect attacks and environmental stress. The nicotine produced by tobacco is concerned with environmental factors and hormone levels. Earlier studies reported that COR could induce plant alkaloid accumulation [63], and this induction effect mainly occurs via the activation of *NtMYC2*, a key transcription factor in JA signaling that the regulates expression of limiting enzyme during the nicotine biosynthesis process [64,65]. In addition, we found choline levels were significantly increased in COR-treated plants under osmotic stress. Choline is an important intermediate for the biosynthesis of cell membrane components (e.g., phosphorylcholine). Decreases in choline content of NaCl-treated tobacco plants suggested that salinity inhibited the synthesis of membrane or facilitated degradation of membrane [66]. Choline is also the precursor of glycine betaine, a well-characterized compatible solute involved in osmotic stress. Zhang et al. indicated that enhanced biosynthesis of choline improved *Arabidopsis* osmotic stress tolerance via promoting osmoprotectant accumulation [67].

### 4.5. Increased Levels of Amino Acids in PEG-Stressed Tobacco Plants May Be an Indicator of Tissue Damage

The imposition of abiotic stress increases the accumulation of amino acids in many plants [68,69,70,71]. The increased accumulation of amino acids during drought stress is, in some cases, connected with contrary effects on nitrogen metabolism and the stress-induced hydrolysis of proteins [72,73,74]. In the present work, most of the amino acids exhibited increased accumulation in control tobacco leaves under PEG stress compared to that COR treatment. Diaz et al. suggested that the relative content of some amino acids (e.g., leucine, isoleucine) may serve as chemical markers to discriminate senescence as they were correlated to leaf yellowing [75]. However, it is interesting to have found that a higher level of proline and GABA was observed in seedlings pre-treated with COR. Proline acts as a non-toxic osmolyte and mitigates drought stress in plants by quenching ROS and maintaining the stabilization of cell membranes [76]. Previous studies have shown that COR promoted proline content in soybean [13], cauliflower [14], rice [17], and maize [77] during abiotic stresses. GABA, a four-carbon non-protein amino acid, has an active role in plant physiological process, such as regulating cellar pH, contributing to nitrogen/carbohydrate balance, and protecting against oxidative stress [78]. Shang et al. revealed that peach fruit pre-treated with GABA reduced chilling injury by induction of endogenous GABA and proline accumulation [79]. Combined with our physiological and biochemical data on osmotic stress (e.g., FW, DW, O_2_^•−^, H_2_O_2_), we conclude that the increase in amino acids (except for proline and GABA) is a biomarker of osmotic stress, rather than an adaption strategy.

## 5. Conclusions

Drought stress can cause severe problems in the phenotype, physiology, and metabolism of a plant during its life cycle. The use of a harmless plant growth regulator to protect crops from damage caused by stress, especially drought stress, is of great economic value. In this study, we first determined the effect of a micro-dose of COR on tobacco under PEG-simulated drought stress. From this integrative work, we conclude that, under osmotic stress, COR could regulate the activities of antioxidant enzymes to prevent ROS accumulation and lipid peroxidation. Several primary or secondary metabolites, such as carbohydrates, organic acids, and nitrogen-containing compounds, that might contribute to osmotic stress tolerance were specifically accumulated in COR treatment. This study highlights the molecular mechanism of COR-mediated tolerance to osmotic stress in tobacco and broadly in crop plants. The specifically accumulated metabolites, together with the metabolic network identified here, provide valuable clues for the further studies of COR-induced stress resistance in plants.

## Figures and Tables

**Table 1:** Effect of COR on biomass of tobacco seedlings under PEG treatment.

Treatment	FW (g/Plant)	DW (g/Plant)
CK	17.06 ± 0.59 a	1.72 ± 0.04 a
COR	17.20 ± 0.49 a	1.72 ± 0.03 a
PEG	11.27 ± 0.46 c	1.43 ± 0.05 c
PEG+COR	14.09 ± 0.51 b	1.60 ± 0.04 b

Values are the means ± SDs (n = 5), different letters within a column indicate significantly different at *p* < 0.05. FW = fresh weight; DW = dry weight.

**Table 2:** List of the significantly changed metabolites between COR-treated and non-treated tobacco seedlings under normal growth conditions and PEG stress. VIP = variable importance in projection; ESI= electrospray ionization source.

Mode	Metabolite	VIP	FC	*p* Value
Sugar and sugar derivatives				
ESI (−)	D-Allose ^1^	2.22	2.07	2.0 × 10^−3^
ESI (+)	Galactinol ^1^	2.70	2.15	9.0 × 10^−7^
ESI (−)	α-D-Glucose ^2^	1.56	2.13	6.3 × 10^−4^
ESI (−)	D-Fructose ^2^	1.68	2.33	2.9 × 10^−4^
ESI (+)	D-Mannose ^2^	2.21	6.62	1.7 × 10^−8^
ESI (−)	D-Quinovose ^2^	1.80	2.48	2.4 × 10^−7^
ESI (−)	Maltitol ^2^	2.16	3.69	2.7 × 10^−7^
ESI (+)	Myo-Inositol ^2^	1.57	2.60	3.6 × 10^−8^
Organic acids				
ESI (−)	α-Ketoglutarate ^1^	2.16	2.08	2.9 × 10^−2^
ESI (−)	Chlorogenic acid ^1^	1.84	1.59	7.2 × 10^−6^
ESI (+)	Citrate ^1^	2.28	1.79	2.0 × 10^−4^
ESI (−)	α-Ketoglutarate ^2^	1.58	2.57	4.5 × 10^−3^
ESI (−)	Citrate ^2^	2.06	3.45	4.8 × 10^−6^
ESI (−)	Glyceric acid ^2^	1.30	1.76	5.0 × 10^−4^
ESI (−)	Quinic acid ^2^	2.55	6.32	2.1 × 10^−7^
Amino acids				
ESI (+)	D-Proline ^2^	1.92	4.10	5.1 × 10^−8^
ESI (+)	GABA ^2^	1.19	1.80	4.5 × 10^−5^
ESI (+)	L-Arginine ^2^	1.53	0.38	2.0 × 10^−5^
ESI (+)	L-Asparagine ^2^	1.52	0.39	1.9 × 10^−5^
ESI (+)	L-Histidine ^2^	1.18	0.55	7.3 × 10^−5^
ESI (+)	L-Isoleucine ^2^	1.98	0.22	1.7 × 10^−9^
ESI (+)	L-Leucine ^2^	1.95	0.23	2.4 × 10^−10^
ESI (−)	L-Lysine ^2^	1.91	0.33	1.5 × 10^−3^
ESI (−)	L-Norleucine ^2^	2.61	0.15	8.0 × 10^−7^
ESI (−)	L-Threonine ^2^	1.94	0.27	5.2 × 10^−4^
ESI (−)	L-Tryptophan ^2^	2.41	0.20	1.7 × 10^−9^
Others				
ESI (+)	Nicotinamide ^1^	3.25	3.12	4.8 × 10^−4^
ESI (+)	2-Ethoxyethanol ^2^	1.17	0.52	1.0 × 10^−3^
ESI (+)	4-Hydroxybutanoic acid lactone ^2^	1.07	0.61	4.7 × 10^−4^
ESI (+)	Choline ^2^	1.36	2.07	1.1 × 10^−6^
ESI (+)	L-Nicotine ^2^	1.29	2.07	7.4 × 10^−4^
ESI (+)	Nicotinamide ^2^	1.45	2.89	5.9 × 10^−3^
ESI (+)	Phosphorylcholine ^2^	1.43	2.46	4.5 × 10^−3^
ESI (+)	Trigonelline ^2^	1.04	1.88	9.5 × 10^−3^

Number “^1^” and “^2^” represent the significantly changed metabolites identified under normal growth conditions and PEG stress, respectively. ESI (−) and ESI (+) represent positive ion mode and negative ion mode, respectively.

**Table 3:** KEGG pathways including more than ≥2 significantly changed metabolites affected by COR.

ID	KEGG Pathway	Compounds
map00970	Aminoacyl-tRNA biosynthesis	C00152, C00135, C00062, C00047, C00407, C00123, C00188, C00078
map00052	Galactose metabolism	C00267, C01235, C00137, C00159
map00260	Glycine, serine, and threonine metabolism	C00114, C00258, C00188, C00078
map00290	Valine, leucine, and isoleucine biosynthesis	C00123, C00188, C00407
map00250	Alanine, aspartate, and glutamate metabolism	C00152, C00026, C00334
map00966	Glucosinolate biosynthesis	C00078, C00123, C00407
map00220	Arginine biosynthesis	C00026, C00062
map00051	Fructose and mannose metabolism	C00267, C00159
map00020	Citrate cycle (tricarboxylic acid (TCA) cycle)	C00158, C00026
map00330	Arginine and proline metabolism	C00062, C00334
map00280	Valine, leucine, and isoleucine degradation	C00407, C00123
map00564	Glycerophospholipid metabolism	C00114, C00588

KEGG database matched results, C00026: α-ketoglutarate; C00047: L-lysine; C00062: L-arginine; C00078: L-tryptophan; C00114: choline; C00123: L-leucine; C00135: L-histidine; C00137: myo-inositol; C00152: L-asparagine; C00158: citric acid; C00159: D-mannose; C00188: L-threonine; C00258: glyceric acid; C00267: α-D-glucose; C00334: GABA; C00407: L-isoleucine; C00588: phosphorylcholine; C01235: galactinol.

## Supplementary Materials

The following are available online at https://www.mdpi.com/2218-273X/10/1/99/s1, Figure S1: Score plot of partial least square discriminant analysis of metabolites in tobacco leaves between the COR and the control treatment under normal growth conditions (A, B) and PEG stress (C, D). CK, normal growth conditions; COR, pretreatment with 1 nM COR; PEG, osmotic stress induced by PEG without COR treatment; PEG+COR, pretreatment with 1 nM COR under PEG stress.

## References

[1] Hopkins A., Del Prado A. (2007). Implications of climate change for grassland in Europe: Impacts, adaptations and mitigation options: A review. Grass Forage Sci..

[2] Blum A. (1996). Crop responses to drought and the interpretation of adaptation. Plant Growth Regul..

[3] Yu C.Q., Huang X., Chen H., Huang G.R., Ni S.Q., Wright J.S., Hall J., Ciais P., Zhang J., Xiao Y.C. (2018). Assessing the Impacts of Extreme Agricultural Droughts in China Under Climate and Socioeconomic Changes. Earth Future.

[4] Kang Z.Y., Babar M.A., Khan N., Guo J., Khan J., Islam S., Shrestha S., Shahi D. (2019). Comparative metabolomic profiling in the roots and leaves in contrasting genotypes reveals complex mechanisms involved in post-anthesis drought tolerance in wheat. PLoS ONE.

[5] Anjum S.A., Xie X.Y., Wang L.C., Saleem M.F., Man C., Lei W. (2011). Morphological, physiological and biochemical responses of plants to drought stress. Afr. J. Agric. Res..

[6] Bender C., Palmer D., PenalozaVazquez A., Rangaswamy V., Ullrich M. (1996). Biosynthesis of coronatine, a thermoregulated phytotoxin produced by the phytopathogen *Pseudomonas syringae*. Arch. Microbiol..

[7] Tamogami S., Kodama O. (2000). Coronatine elicits phytoalexin production in rice leaves (*Oryza sativa* L.) in the same manner as jasmonic acid. Phytochemistry.

[8] Uppalapati S.R., Ayoubi P., Weng H., Palmer D.A., Mitchell R.E., Jones W., Bender C.L. (2005). The phytotoxin coronatine and methyl jasmonate impact multiple phytohormone pathways in tomato. Plant J..

[9] Kenyon J.S., Turner J.G. (1992). The stimulation of ethylene synthesis in nicotiana-tabacum leaves by the phytotoxin coronatine. Plant Physiol..

[10] Zhang Z., Yang F., Li B., Eneji A.E., Li J., Duan L., Wang B., Li Z., Tian X. (2009). Coronatine-induced lateral-root formation in cotton (*Gossypium hirsutum*) seedlings under potassium-sufficient and -deficient conditions in relation to auxin. J. Plant Nutr. Soil Sci..

[11] Feys B.J.F., Benedetti C.E., Penfold C.N., Turner J.G. (1994). Arabidopsis mutants selected for resistance to the phytotoxin coronatine are male-sterile, insensitive to methyl jasmonate, and resistant to a bacterial pathogen. Plant Cell.

[12] Xie Z.X., Duan L.S., Tian X.L., Wang B.M., Eneji A.E., Li Z.H. (2008). Coronatine alleviates salinity stress in cotton by improving the antioxidative defense system and radical-scavenging activity. J. Plant Physiol..

[13] Hao L., Wang Y., Zhang J., Xie Y., Zhang M., Duan L., Li Z. (2013). Coronatine enhances drought tolerance via improving antioxidative capacity to maintaining higher photosynthetic performance in soybean. Plant Sci..

[14] Wu H., Wu X., Li Z., Duan L., Zhang M. (2012). Physiological Evaluation of Drought Stress Tolerance and Recovery in Cauliflower (*Brassica oleracea* L.) Seedlings Treated with Methyl Jasmonate and Coronatine. J. Plant Growth Regul..

[15] Zhou Y., Zhang M., Li J., Li Z., Tian X., Duan L. (2015). Phytotoxin coronatine enhances heat tolerance via maintaining photosynthetic performance in wheat based on Electrophoresis and TOF-MS analysis. Sci. Rep..

[16] Wang L., Chen W.J., Wang Q., Eneji A.E., Li Z.H., Duan L.S. (2009). Coronatine Enhances Chilling Tolerance in Cucumber (*Cucumis sativus* L.) Seedlings by Improving the Antioxidative Defence System. J. Agron. Crop Sci..

[17] Gao W., Yu C.X., Ai L., Zhou Y.Y., Duan L.S. (2019). Gene Expression Profiles Deciphering the Pathways of Coronatine Alleviating Water Stress in Rice (*Oryza sativa* L.) Cultivar Nipponbare (Japonica). Int. J. Mol. Sci..

[18] Wen W.W., Li K., Alseekh S., Omranian N., Zhao L.J., Zhou Y., Xiao Y.J., Jin M., Yang N., Liu H.J. (2015). Genetic Determinants of the Network of Primary Metabolism and Their Relationships to Plant Performance in a Maize Recombinant Inbred Line Population. Plant Cell.

[19] Go E.P. (2010). Database Resources in Metabolomics: An Overview. J. Neuroimmune Pharm..

[20] Simo C., Ibanez C., Valdes A., Cifuentes A., Garcia-Canas V. (2014). Metabolomics of Genetically Modified Crops. Int. J. Mol. Sci..

[21] Scalabrin E., Radaelli M., Rizzato G., Bogani P., Buiatti M., Gambaro A., Capodaglio G. (2015). Metabolomic analysis of wild and transgenic Nicotiana langsdorffii plants exposed to abiotic stresses: Unraveling metabolic responses. Anal. Bioanal. Chem..

[22] Mibei E.K., Owino W.O., Ambuko J., Giovannoni J.J., Onyango A.N. (2018). Metabolomic analyses to evaluate the effect of drought stress on selected African Eggplant accessions. J. Sci. Food Agric..

[23] Tian J., Jiang F.L., Wu Z. (2015). The apoplastic oxidative burst as a key factor of hyperhydricity in garlic plantlet in vitro. Plant Cell Tiss. Organ Cult..

[24] Scarpeci T.E., Zanor M.I., Carrillo N., Mueller-Roeber B., Valle E.M. (2008). Generation of superoxide anion in chloroplasts of Arabidopsis thaliana during active photosynthesis: A focus on rapidly induced genes. Plant Mol. Biol..

[25] Daudi A., Cheng Z.Y., O’Brien J.A., Mammarella N., Khan S., Ausubel F.M., Bolwell G.P. (2012). The Apoplastic Oxidative Burst Peroxidase in Arabidopsis Is a Major Component of Pattern-Triggered Immunity. Plant Cell.

[26] Bradford M.M. (1976). Rapid and sensitive method for quantitation of microgram quantities of protein utilizing principle of protein-dye binding. Anal. Biochem..

[27] Li Y.C., Ma Y.Y., Zhang T.T., Bi Y., Wang Y., Prusky D. (2019). Exogenous polyamines enhance resistance to Alternaria alternata by modulating redox homeostasis in apricot fruit. Food Chem..

[28] Adusumilli R., Mallick P., Comai L., Katz J.E., Mallick P. (2017). Data Conversion with ProteoWizard msConvert. Proteomics: Methods and Protocols.

[29] Patti G.J., Tautenhahn R., Siuzdak G. (2012). Meta-analysis of untargeted metabolomic data from multiple profiling experiments. Nat. Protoc..

[30] Smith C.A., Want E.J., O’Maille G., Abagyan R., Siuzdak G. (2006). XCMS: Processing mass spectrometry data for metabolite profiling using Nonlinear peak alignment, matching, and identification. Anal. Chem..

[31] Fan W.Q., Ge G.T., Liu Y.H., Wang W., Liu L.Y., Jia Y.S. (2018). Proteomics integrated with metabolomics: Analysis of the internal causes of nutrient changes in alfalfa at different growth stages. BMC Plant Biol..

[32] Chong J., Soufan O., Li C., Caraus I., Li S.Z., Bourque G., Wishart D.S., Xia J.G. (2018). MetaboAnalyst 4.0: Towards more transparent and integrative metabolomics analysis. Nucleic Acids Res..

[33] Xia J.G., Wishart D.S. (2010). MSEA: A web-based tool to identify biologically meaningful patterns in quantitative metabolomic data. Nucleic Acids Res..

[34] Yu W.W., Wang Z., Zhang K.L., Chi Z.X., Xu T., Jiang D.L., Chen S., Li W.X., Yang X.Y., Zhang X. (2019). One-Carbon Metabolism Supports S-Adenosylmethionine and Histone Methylation to Drive Inflammatory Macrophages. Mol. Cell.

[35] McCord J.M. (2000). The evolution of free radicals and oxidative stress. Am. J. Med..

[36] Upadhyaya H., Khan M.H., Panda S.K. (2007). Hydrogen peroxide induces oxidative stress in detached leaves of *Oryza sativa* L.. Gen. Appl. Plant Physiol..

[37] Gill S.S., Tuteja N. (2010). Reactive oxygen species and antioxidant machinery in abiotic stress tolerance in crop plants. Plant Physiol. Biochem..

[38] Ai L., Li Z.H., Xie Z.X., Tian X.L., Eneji A.E., Duan L.S. (2008). Coronatine alleviates polyethylene glycol-induced water stress in two rice (*Oryza sativa* L.) cultivars. J. Agron. Crop Sci..

[39] Li X.W., Shen X.F., Li J.M., Eneji A.E., Li Z.H., Tian X.L., Duan L.S. (2010). Coronatine Alleviates Water Deficiency Stress on Winter Wheat Seedlings. J. Integr. Plant Biol..

[40] Gupta A.K., Kaur N. (2005). Sugar signalling and gene expression in relation to carbohydrate metabolism under abiotic stresses in plants. J. Biosci..

[41] Kumari A., Parida A.K. (2018). Metabolomics and network analysis reveal the potential metabolites and biological pathways involved in salinity tolerance of the halophyte Salvadora persica. Environ. Exp. Bot..

[42] Rolland F., Baena-Gonzalez E., Sheen J. (2006). Sugar sensing and signaling in plants: Conserved and novel mechanisms. Annu. Rev. Plant Biol..

[43] Liu F.L., Jensen C.R., Andersen M.N. (2004). Drought stress effect on carbohydrate concentration in soybean leaves and pods during early reproductive development: Its implication in altering pod set. Field Crop. Res..

[44] Couee I., Sulmon C., Gouesbet G., El Amrani A. (2006). Involvement of soluble sugars in reactive oxygen species balance and responses to oxidative stress in plants. J. Exp. Bot..

[45] Chakraborty K., Bishi S.K., Singh A.L., Zala P.V., Mahatma M.K., Kalariya K.A., Jat R.A. (2018). Rapid induction of small heat shock proteins improves physiological adaptation to high temperature stress in peanut. J. Agron. Crop Sci..

[46] Nam K.H., Shin H.J., Pack I.S., Park J.H., Kim H.B., Kim C.G. (2016). Metabolomic changes in grains of well-watered and drought-stressed transgenic rice. J. Sci. Food Agric..

[47] Li Z., Yu J.J., Peng Y., Huang B.R. (2017). Metabolic pathways regulated by abscisic acid, salicylic acid and -aminobutyric acid in association with improved drought tolerance in creeping bentgrass (Agrostis stolonifera). Physiol. Plant..

[48] Wheeler G.L., Jones M.A., Smirnoff N. (1998). The biosynthetic pathway of vitamin C in higher plants. Nature.

[49] Smirnoff N., Wheeler G.L. (2000). Ascorbic acid in plants: Biosynthesis and function. Crit. Rev. Biochem. Mol. Biol..

[50] Sengupta S., Mukherjee S., Goswami L., Sangma S., Mukherjee A., Mukherjee R., Roy N., Basak P., Majumder A.L. (2012). Manipulation of inositol metabolism for improved plant survival under stress: A “network engineering approach”. J. Plant Biochem. Biotechnol..

[51] Smirnoff N., Cumbes Q.J. (1989). Hydroxyl radical scavenging activity of compatible solutes. Phytochemistry.

[52] Timpa J.D., Burke J.J., Quisenberry J.E., Wendt C.W. (1986). Effects of water-stress on the organic-acid and carbohydrate compositions of cotton plants. Plant Physiol..

[53] Zhang W.F., Gong Z.H., Wu M.B., Chan H.L., Yuan Y.J., Tang N., Zhang Q., Miao M.J., Chang W., Li Z. (2019). Integrative comparative analyses of metabolite and transcript profiles uncovers complex regulatory network in tomato (*Solanum lycopersicum* L.) fruit undergoing chilling injury. Sci. Rep..

[54] Wu X.P., Dai H.L., Liu L.L., Xu C., Yin Y.X., Yi J.L., Bielec M.D., Han Y.C., Li S.P. (2019). Citrate reduced oxidative damage in stem cells by regulating cellular redox signaling pathways and represent a potential treatment for oxidative stress-induced diseases. Redox Biol..

[55] Sung J., Lee S., Lee Y., Ha S., Song B., Kim T., Waters B.M., Krishnan H.B. (2015). Metabolomic profiling from leaves and roots of tomato (*Solanum lycopersicum* L.) plants grown under nitrogen, phosphorus or potassium-deficient condition. Plant Sci..

[56] Li Z., Cheng B.Z., Yong B., Liu T., Peng Y., Zhang X.Q., Ma X., Huang L.K., Liu W., Nie G. (2019). Metabolomics and physiological analyses reveal beta-sitosterol as an important plant growth regulator inducing tolerance to water stress in white clover. Planta.

[57] Berglund T., Ohlsson A.B. (1995). Defensive and secondary metabolism in plant tissue cultures, with special reference to nicotinamide, glutathione and oxidative stress. Plant Cell Tiss. Organ Cult..

[58] Azooz M. (1560). Proteins, sugars and ion leakage as a selection criterion for the salt tolerance of three sorghum cultivars at seedling stage grown under NaCl and nicotinamide. Int. J. Agric. Biol..

[59] Tramontano W.A., Jouve D. (1997). Trigonelline accumulation in salt-stressed legumes and the role of other osmoregulators as cell cycle control agents. Phytochemistry.

[60] Cho Y.K., Lightfoot D.A., Wood A.J. (1999). Trigonelline concentrations in salt stressed leaves of cultivated Glycine max. Phytochemistry.

[61] Rajasekaran L.R., Aspinall D., Jones G.P., Paleg L.G. (2001). Stress metabolism. IX. Effect of salt stress on trigonelline accumulation in tomato. Can. J. Plant Sci..

[62] Cho Y., Njiti V.N., Chen X., Lightfoot D.A., Wood A.J. (2003). Trigonelline concentration in field-grown soybean in response to irrigation. Biol. Plant..

[63] Xie Z.X., Duan L.S., Li Z.H., Wang X.D., Liu X.J. (2015). Dose-Dependent Effects of Coronatine on Cotton Seedling Growth Under Salt Stress. J. Plant Growth Regul..

[64] Shoji T., Ogawa T., Hashimoto T. (2008). Jasmonate-induced nicotine formation in tobacco is mediated by tobacco COI1 and JAZ genes. Plant Cell Physiol..

[65] Shoji T., Hashimoto T. (2011). Tobacco MYC2 Regulates Jasmonate-Inducible Nicotine Biosynthesis Genes Directly and By Way of the NIC2-Locus ERF Genes. Plant Cell Physiol..

[66] Zhang J.T., Zhang Y., Du Y.Y., Chen S.Y., Tang H.R. (2011). Dynamic Metabonomic Responses of Tobacco (Nicotiana tabacum) Plants to Salt Stress. J. Proteome Res..

[67] Zhang H.M., Murzello C., Sun Y., Kim M.S., Xie X.T., Jeter R.M., Zak J.C., Dowd S.E., Pare P.W. (2010). Choline and Osmotic-Stress Tolerance Induced in Arabidopsis by the Soil Microbe Bacillus subtilis (GB03). Mol. Plant-Microbe Interact..

[68] Nikiforova V.J., Bielecka M., Gakiere B., Krueger S., Rinder J., Kempa S., Morcuende R., Scheible W.R., Hesse H., Hoefgen R. (2006). Effect of sulfur availability on the integrity of amino acid biosynthesis in plants. Amino Acids.

[69] Lugan R., Niogret M.F., Leport L., Guegan J.P., Larher F.R., Savoure A., Kopka J., Bouchereau A. (2010). Metabolome and water homeostasis analysis of Thellungiella salsuginea suggests that dehydration tolerance is a key response to osmotic stress in this halophyte. Plant J..

[70] Sehgal A., Sita K., Bhandari K., Kumar S., Kumar J., Prasad P.V.V., Siddique K.H.M., Nayyar H. (2019). Influence of drought and heat stress, applied independently or in combination during seed development, on qualitative and quantitative aspects of seeds of lentil (Lens culinaris Medikus) genotypes, differing in drought sensitivity. Plant Cell Environ..

[71] Jia H., Wang L., Li J., Sun P., Lu M., Hu J. (2019). Comparative metabolomics analysis reveals different metabolic responses to drought in tolerant and susceptible poplar species. Physiol. Plant..

[72] Krasensky J., Jonak C. (2012). Drought, salt, and temperature stress-induced metabolic rearrangements and regulatory networks. J. Exp. Bot..

[73] Widodo, Patterson J.H., Newbigin E., Tester M., Bacic A., Roessner U. (2009). Metabolic responses to salt stress of barley (*Hordeum vulgare* L.) cultivars, Sahara and Clipper, which differ in salinity tolerance. J. Exp. Bot..

[74] Roessner U., Patterson J.H., Forbes M.G., Fincher G.B., Langridge P., Bacic A. (2006). An investigation of boron toxicity in barley using metabolomics. Plant Physiol..

[75] Diaz C., Purdy S., Christ A., Morot-Gaudry J.F., Wingler A., Masclaux-Daubresse C.L. (2005). Characterization of markers to determine the extent and variability of leaf senescence in Arabidopsis. A metabolic profiling approach. Plant Physiol..

[76] Hayat S., Hayat Q., Alyemeni M.N., Wani A.S., Pichtel J., Ahmad A. (2012). Role of proline under changing environments A review. Plant Signal. Behav..

[77] Wang B.Q., Li Z.H., Eneji A.E., Tian X.L., Zhai Z.X., Li J.M., Duan L.S. (2008). Effects of coronatine on growth, gas exchange traits, chlorophyll content, antioxidant enzymes and lipid peroxidation in maize (*Zea mays* L.) seedlings under simulated drought stress. Plant Prod. Sci..

[78] Bouche N., Fromm H. (2004). GABA in plants: Just a metabolite?. Trends Plant Sci..

[79] Shang H.T., Cao S.F., Yang Z.F., Cai Y.T., Zheng Y.H. (2011). Effect of Exogenous gamma-Aminobutyric Acid Treatment on Proline Accumulation and Chilling Injury in Peach Fruit after Long-Term Cold Storage. J. Agric. Food Chem..

